# Hospitalizations Are Increasing in Distal Upper Extremity Fractures

**DOI:** 10.7759/cureus.38870

**Published:** 2023-05-11

**Authors:** Jeffrey B Brown, Jacob Albers, Demetrios I Tsirukis, Daniel J Strebig, Ambuj Kumar, Jeremy Hreha

**Affiliations:** 1 Department of Medical Education, University of South Florida Morsani College of Medicine, Tampa, USA; 2 Department of Medical Education, Midwestern University Chicago College of Osteopathic Medicine, Downers Grove, USA; 3 Department of Internal Medicine/Biostatistics and Epidemiology, University of South Florida Morsani College of Medicine, Tampa, USA; 4 Department of Orthopedic Surgery, Lehigh Valley Health Network, Allentown, USA

**Keywords:** distal upper extremity, upper extremity fractures, resource allocation, hospitalization, hand, wrist, fracture

## Abstract

Introduction

Distal upper extremity (DUE) fractures are common and include bony fractures of the wrist, hand, and finger. DUE fractures can require hospital admission for clinical observation or surgical fixation. The trend of hospitalization rate for these injuries may more accurately predict future staffing needs, required resources, and expected revenue for orthopedic surgery hand services. The purpose of this study is to determine the trend of hospitalization percentage from 2009 to 2018 for patients presenting to the United States (US) emergency departments (EDs) with DUE fractures.

Methods

The National Electronic Injury Surveillance System (NEISS) was utilized to collect data from 138,700 patients with wrist, hand, or finger fractures presenting to the US EDs between 2009 and 2018. A total of 752 patients were excluded for ages less than two years old or no sex entry. The unadjusted and adjusted (age, sex, race, and fracture location) hospitalization rates across years were evaluated using binary logistic regression.

Results

From 2009 to 2018, 137,948 DUE fractures were reported, of which 4749 (3.4%) were hospitalized. Wrist fractures accounted for the highest amount (2953) and the highest proportion of hospitalized patients (62.2%). Higher hospitalization rates were seen among patients 40 years and older (p < 0.05). Together, the DUE fracture hospitalization rate increased significantly (p < 0.05) in 2016 (OR = 1.215, 95% CI = 1.070-1.380), 2017 (OR = 1.154, 95% CI = 1.016-1.311), and 2018 (OR = 1.154, 95% CI = 1.279-1.638) from 2009. The adjusted results showed hospitalization rate statistically increased (p < 0.05) in 2016 (OR = 1.184, 95% CI = 1.040-1.346) and 2018 (OR = 1.389, 95% CI = 1.225-1.575) compared to 2009. An inconsistent increase in hospitalization rate was seen across locations of fracture: wrist (2012, 2013, 2018), hand (2018), and finger (2016, 2018).

Conclusions

The hospitalization rate of patients with DUE fractures increased in 2016 and 2018 from 2009. These data may predict a need to increase future staffing and resources for orthopedic surgery hand services as hospitals resume pre-pandemic practices.

## Introduction

Distal upper extremity (DUE) injuries, including those of the wrist, hand, and finger, account for about 20% of complaints that present to emergency departments (EDs) in the United States (US) [1]. Bony fractures are the second most common type of DUE injury, behind soft tissue contusion [2]. Despite the large number of these injuries presenting to the ED, many are not routinely hospitalized [2]. Improper management of these injuries can lead to disability, chronic pain, and decreased quality of life [3]. While some of these fractures are treated in the outpatient setting, those patients who are admitted to the hospital generally require an operative intervention of bony and soft tissue injuries or have an unrelated reason for hospitalization [4].

Orthopedic hand services treat the majority of hospitalized upper extremity fracture patients. Thus, changes in the hospitalization rate of these patients directly affect resource usage, workload, and staffing management for these services. Existing literature in the US has described the number of DUE fractures presenting to EDs. However, current trends of hospitalizations due to DUE orthopedic injuries are not presently published. Understanding these trends may allow us to speculate staffing needs and expected revenue for orthopedic hand services. The purpose of this study is to determine the trend of hospitalization percentage from 2009 to 2018 for patients presenting to the US EDs with DUE fractures.

## Materials and methods

Study design

A multi-center, retrospective cohort study was performed following the Strengthening the Reporting of Observational Studies in Epidemiology (STROBE) guidelines. This study was exempt from an institutional review board as the data were open-source and de-identified.

Patients

Patient data were collected from the National Electronic Injury Surveillance System (NEISS) database, which contains de-identified patient information collected from over 100 EDs across the US [5]. The clinical sites from which the data are compiled serve as a representative sample of national data. All consecutive patients with a primary injury of wrist, hand, or finger fracture in the years 2009 to 2018 were eligible for study inclusion. Patients were excluded from the study if they were less than two years old or had no sex entry.

Primary outcome

The primary outcome measure was the hospitalization rate for patients presenting to EDs for a primary diagnosis of wrist, hand, or finger fracture. Wrist fractures included those of the distal radius, distal ulna, and carpal bones. Hand fractures included those of the metacarpal bones, and finger fractures included those of the phalanges. All patients categorized as hospitalized were admitted to the ED, transferred and admitted to a separate institution, or admitted for observation. The comparison arm of the study included patients who had primary wrist, hand, or finger fractures and were evaluated but not admitted to the hospital.

Secondary outcomes

The secondary outcomes of our study investigated hospitalization trends in wrist, hand, and finger fractures according to sex, age, and fracture location. Sex analysis compared male and female hospitalization rates. Age analysis compared patients in three categories: below 18 years, 18-49 years, and 50 years and older. These age ranges were chosen to compare differences in the disposition of pediatric patients, younger adults, and older adults. Additionally, these age groups were similar to those of previous studies, increasing the ease of direct comparison [6,7]. Fracture location subgroup analysis compared hospitalization rates in wrist, hand, and finger fractures.

Statistical analysis

Demographic variables and patient characteristics were reported as mean and standard deviation for continuous variables and as percentages for categorical variables. The crude and adjusted trends in hospitalization rates due to fractures were assessed using binary logistic regression and were reported as odds ratios with 95% confidence intervals (CI). Statistical significance was set at 5% for all comparisons. All analyses were performed using the IBM SPSS statistical analysis package version 29 (IBM Corp., Armonk, NY).

## Results

Data were collected for 138,700 patients. A total of 752 patients were excluded: 750 for ages less than two years and two for no sex entry. A total of 137,948 patients with wrist, hand, or finger fractures were included in the primary analysis (Table 1). The average age was 27.8 (±25.9) years, and the majority of patients were male (65.3%). Across the 10 years, only 3.4% of patients were hospitalized (n = 4749). Patients with wrist fractures were hospitalized 5.8% of the time and accounted for the majority of DUE fracture hospitalizations (n = 2953). Patients with hand fractures were hospitalized 2.0% of the time (n = 634), while patients with finger fractures were hospitalized 2.1% of the time (n = 1162). Patients with finger fractures were most commonly presented to the ED (n = 54,992, 39.9%), followed by wrist fractures (n = 51,037, 37.0%) and hand fractures (n = 31,919, 23.1%).

**Table 1:** Distal upper extremity fracture demographics and characteristics

Year	2009	2010	2011	2012	2013	2014	2015	2016	2017	2018	P-value
	(N = 15,306)	(N = 15,511)	(N = 14,863)	(N = 14,181)	(N = 13,215)	(N = 12,422)	(N = 12,108)	(N = 13,530)	(N = 13,939)	(N = 12,873)	
Age (years)											
Mean (standard deviation)	27 (±25)	27 (±26)	27 (±25)	28 (±26)	28 (±25)	28 (±25)	29 (±26)	28 (±27)	28 (±26)	29 (±28)	<0.001
Sex, No. (%)											
Male	9930 (65)	10,140 (65)	9639 (65)	9209 (65)	8594 (65)	8091 (65)	7781 (64)	8478 (63)	8640 (62)	8024 (62)	<0.001
Female	5376 (35)	5371 (35)	5224 (35)	4972 (35)	4621 (35)	4331 (35)	4327 (36)	5052 (37)	5299 (38)	4849 (38)	
Race, No. (%)											
White	7808 (51)	7439 (48)	7461 (50)	7320 (52)	6360 (48)	5570 (49)	4865 (40)	5480 (41)	5971 (43)	5629 (44)	<0.001
African American	1844 (12)	2071 (13)	2048 (14)	1970 (14)	1769 (13)	1728 (14)	1610 (13)	1791 (13)	1896 (14)	1739 (14)	
Asian	0 (0)	186 (12)	204 (1)	186 (1)	156 (1)	155 (1)	147 (1)	170 (1)	191 (1)	138 (1)	
American Indian/Alaska Native	0 (0)	32 (0)	50 (0)	34 (0)	34 (0)	30 (0)	33 (0)	40 (0)	48 (0)	47 (0)	
Native Hawaiin/Pacific Islander	0 (0)	7 (0)	10 (0)	13 (0)	13 (0)	14 (0)	14 (0)	16 (0)	16 (0)	11 (0)	
Other	1265 (8)	1280 (8)	1119 (8)	1032 (7)	1109 (8)	895 (7)	662 (6)	929 (7)	916 (7)	593 (5)	
Not stated	4389 (29)	4496 (29)	3971 (27)	3626 (26)	3774 (29)	4030 (32)	4777 (40)	5104 (38)	4901 (35)	4716 (37)	
Fracture site, No. (%)											
Wrist	5879 (38)	5691 (37)	5346 (36)	4823 (34)	4667 (35)	4290 (35)	4328 (36)	5176 (38)	5576 (40)	5261 (41)	<0.001
Hand	3518 (23)	3603 (23)	3529 (24)	3440 (24)	3195 (24)	3051 (25)	2844 (23)	2993 (22)	2979 (21)	2767 (21)	
Finger	5909 (39)	6217 (40)	5988 (40)	5918 (42)	5353 (41)	5081 (41)	4936 (41)	5361 (40)	5384 (39)	4845 (38)	

Primary outcome

The average rate of hospitalization for DUE fractures in the study period was 3.4% (Table 2). Results adjusted for confounding variables (e.g., age, sex, and race) found hospitalization rates for isolated DUE fractures statistically increased (p < 0.05) in 2016 (OR = 1.184, 95% CI = 1.040-1.346) and 2018 (OR = 1.389, 95% CI = 1.225-1.575) compared to 2009.

**Table 2:** Distal upper extremity hospitalization rates compared to 2009

Year	% hospitalized (No.)	P-value	Odds ratio	95% confidence intervals
2009	3.1 (478)			
2010	2.9 (453)	0.275	0.929	0.814-1.060
2011	3.3 (485)	0.508	1.045	0.917-1.190
2012	3.3 (461)	0.639	1.032	0.905-1.177
2013	3.5 (467)	0.080	1.124	0.986-1.282
2014	3.2 (393)	0.973	0.998	0.870-1.145
2015	3.5 (428)	0.160	1.101	0.963-1.260
2016	3.8 (510)	0.010	1.184	1.040-1.346
2017	3.6 (500)	0.064	1.130	0.993-1.286
2018	4.5 (574)	<0.001	1.389	1.225-1.575

Secondary outcomes

Subgroup analysis of fracture location found a statistically significant increase in hospitalization rates for wrist fractures in 2012 (OR = 1.183, 95% CI = 1.003-1.394), 2013 (OR = 1.185, 95% CI = 1.004-1.399), and 2018 (OR = 1.376, 95% CI = 1.178-1.608) compared to 2009. Hand fracture hospitalization rates increased in 2018 (OR = 1.808, 95% CI = 1.282-2.549) compared to 2009. Finger fracture hospitalization rates increased in 2016 (OR = 1.345, 95% CI = 1.046-1.730) and 2018 (OR = 1.325, 95% CI = 1.023-1.716) compared to 2009.

Sex analysis found that male patients diagnosed with DUE fractures had an increased hospitalization rate in 2016 (OR = 1.336, 95% CI = 1.131-1.579) and 2018 (OR = 1.488, 95% CI = 1.261-1.755) compared to 2009. Female patients had an increased hospitalization rate in 2018 (OR = 1.371, 95% CI = 1.138-1.653) compared to 2009.

The majority of patients with DUE fractures were of pediatric age (less than 18 years) (n = 70,896). Younger adults (ages 18-49) (n = 42,271) suffered from DUE fractures more commonly than older adults (ages 50 and older) (n = 24,781). Older adults were hospitalized the most at 9.2% (n = 2281) of the time, followed by younger adults at 2.9% (n = 1212) of the time and pediatric patients at 1.8% (n = 1256) of the time. Older adults were hospitalized at higher rates than younger adults and pediatric patients in all years (p < 0.05). Younger adults were hospitalized at higher rates than pediatric patients in all years (p < 0.05).

## Discussion

Hospitalization rates due to a primary diagnosis of DUE fracture appear to be rising with significant increases in the years 2016 and 2018 compared to the reference year, 2009. Similar trends have been found outside the United States. A 2013 study from the Netherlands found hospitalizations due to wrist fractures increased from 1997 to 2009. The study attributed these trends to increased operative repair methods, citing an increase in the plate and screw fixation constructs for distal radius fractures [8]. Another explanation for the results found in this paper may be the increased adaptation of dedicated trauma operating rooms, which allow treating physicians to treat these fractures more punctually.

Increased hospitalization rates for men (2016 and 2018) and women (2016) were found when compared to 2009. Again, similar trends have been found in the Netherlands [6]. The authors reported wrist fracture hospitalizations more than doubled in women from 30.1 (95% CI = 28.3-31.9) per 100,000 in 1997 to 78.9 (95% CI = 75.1-82.8) per 100,000 in 2009 (p < 0.001), while the total incidence of wrist fractures in women statistically decreased over that time. In men, wrist fracture hospitalizations nearly tripled over the study period from 6.4 (95% CI = 6.0-6.8) per 100,000 in 1997 to 18.4 (95% CI = 17.3-19.5) per 100,000 in 2009, while the total incidence of wrist fractures in men did not statistically change over the study period. These findings illustrate a true increase in overall hospitalization rates in both sexes.

Another notable finding from our study was the increase in hospitalization rate by age. In all years studied, adults aged 50 years and older were hospitalized at higher rates than patients less than 50 years. This may be due to increased operative rates of DUE fractures seen in the US [6]. Mosenthal et al. reported an increase in the rate of open reduction and internal fixation for distal radius fractures between 2007 and 2014 [7]. Additionally, our finding of a positive correlation between age and hospitalization rate in DUE fractures was corroborated by a 2010 study from France, which showed hospitalizations due to wrist fractures increased with age every year from 2002 to 2006 [9]. Age has been shown to be an independent risk factor for both worsened wrist function following certain DUE fractures and the need for surgical repair of DUE fractures [10,11]. Thus, increased aggressive treatment, including hospitalization and surgical repair, of DUE fractures in older adults is important for patient outcomes and is likely contributing to the trends seen in our paper. Furthermore, increased comorbidities seen in older patients may also be contributing to increased hospitalization of this patient population.

This study had several limitations, the largest of which was its reliance on ecological data. The NEISS database is supposed to be a representative sample of national ED data; however, it is impossible to account for unknown biases that impact the selection process for NEISS data collection and how they may influence the overall results. Additionally, the use of the NEISS database may have introduced sample bias as it does not account for patients who presented to private clinics or urgent care centers for care. Errors in diagnosis coding and electronic medical record system specs may under or over-represent DUE fractures being admitted to hospitals as well. Patients with DUE fractures may have been admitted with a different admitting diagnosis, thus underrepresenting the true number of DUE fracture hospitalizations. Another limitation is due to the variability of orthopedic practices' logistics and the presence or absence of an in-hospital orthopedic residency service. For example, some sampled EDs may not have respective orthopedic services to admit patients to, and thus, are more likely to discharge patients with non-surgical treatment. Also, the orthopedic surgeon on-call may not be comfortable treating the fracture and will have the patient obtain outpatient follow-up with a more specialized hand surgeon. Lastly, this analysis does not explain the observed trend of increasing hospitalization rates for DUE fractures.

Nevertheless, to our knowledge, there are no recent studies in the US investigating trends in hospitalization rates due to DUE fractures. The increase in hospitalization attributed to DUE fractures provides us with evidence to plan for resource allocation and staffing, which is critical for future planning.

## Conclusions

This study found a nationwide trend that patients with distal upper extremity fractures, including those of wrist, hand, and finger bones, were increasingly hospitalized from 2009 to 2018. These trends largely remained true for patient sex and fracture location subgroups. In addition, the hospitalization rate for DUE fractures increased with age. While the exact cause of these trends is unknown, some potential causes include increased surgical indications for DUE fractures in patients of all ages or increased elective DUE procedures seen in elderly patients. This trend points toward a future increase in the utilization of orthopedic hand services with increased staffing needs, required hospital resources, and expected revenue for orthopedic hand services.
